# Lung function and radiological findings 1 year after COVID-19: a prospective follow-up

**DOI:** 10.1186/s12931-022-02166-8

**Published:** 2022-09-12

**Authors:** Julia Tarraso, Belen Safont, Juan A. Carbonell-Asins, Estrella Fernandez-Fabrellas, José N. Sancho-Chust, Elsa Naval, Beatriz Amat, Susana Herrera, José A. Ros, Juan J. Soler-Cataluña, Jose A. Rodriguez-Portal, Ada L. Andreu, Margarita Marín, Juan L. Rodriguez-Hermosa, Cruz Gonzalez-Villaescusa, Joan B. Soriano, Jaime Signes-Costa, COVID-FIBROTIC study team, Yolanda García, Natividad Blasco, Antonio Herrera, Alba Mulet, Andrea Ballester, Lucia Fernandez, Antonio Quezada, Elsie Daviana Meneses, Noelia Carrión, Carly Celis, Luis Cabanes, Virginia Molina, Veronica Valentin, Irene López, Elena Solana-Martínez, Mario Aparicio-Vicente, Celia López, Selene Cuenca, Gianna Vargas

**Affiliations:** 1 Pulmonary Department, Hospital Clinico, INCLIVA, Valencia, Spain; 2 Bioinformatics and Biostatistics Unit, INCLIVA, Valencia, Spain; 3 Pulmonary Department, Hospital General, Valencia, Spain; 4 Pulmonary Department, Hospital San Juan, Alicante, Spain; 5 Pulmonary Department, Hospital La Ribera, Alzira, Valencia, Spain; 6 Pulmonary Department, Hospital Vinalopo de Elche, Alicante, Spain; 7 Pulmonary Department, Hospital Dr Peset, Valencia, Spain; 8 Pulmonary Department, Hospital Virgen de la Arrixaca, Murcia, Spain; 9 Pulmonary Department, Hospital Arnau de Vilanova, Valencia, Spain; 10 Pulmonary Department, Hospital Virgen del Rocio, Seville, Spain; 11 Pulmonary Department, Hospital los Arcos, Murcia, Spain; 12 Pulmonary Department, Hospital General, Castellon, Spain; 13 Pulmonary Department, Hospital Clínico San Carlos, Universidad Complutense, Madrid, Spain; 14 Pulmonary Department, Hospital La Princesa, Universidad Autónoma, Madrid, Spain

**Keywords:** Pulmonary sequelae, COVID 19, Lung fibrosis

## Abstract

**Background:**

The coronavirus disease (COVID-19) pandemic has already affected more than 400 million people, with increasing numbers of survivors. These data indicate that a myriad of people may be affected by pulmonary sequelae of the infection. The aim of this study was to evaluate pulmonary sequelae in patients with bilateral COVID-19 pneumonia according to severity 1 year after hospital discharge.

**Methods:**

COVID-FIBROTIC is a multicenter prospective observational cohort study for admitted patients with bilateral COVID-19 pneumonia. Pulmonary functional outcomes and chest computed tomography sequelae were analyzed 12 months after hospital discharge and we classified patients into three groups according to severity. A post hoc analysis model was designed to establish how functional test changed between groups and over time. A multivariable logistic regression model was created to study prognostic factors for lung diffusion impairment and radiological fibrotic-like changes at 12 months.

**Results:**

Among 488 hospitalized patients with COVID-19 pneumonia, 284 patients had completed the entire evaluation at 12 months. Median age was 60.5 ± 11.9 and 55.3% were men. We found between-group differences in male sex, length of hospital stay, radiological involvement and inflammatory laboratory parameters. The functional evaluation of pulmonary sequelae showed that severe patients had statistically worse levels of lung diffusion at 2 months but no between group differences were found in subsequent controls. At 12-month follow up, however, we found impaired lung diffusion in 39.8% unrelated to severity. Radiological fibrotic-like changes at 12 months were reported in 22.7% of patients (102/448), only associated with radiological involvement at admission (OR: 1.55, 95% CI 1.06–2.38; p = 0.02) and LDH (OR: 0.99, 95% CI 0.98–0.99; p = 0.046).

**Conclusion:**

Our data suggest that a significant percentage of individuals would develop pulmonary sequelae after COVID 19 pneumonia, regardless of severity of the acute process.

*Trial registration* clinicaltrials.gov NCT04409275 (June 1, 2020)

**Supplementary Information:**

The online version contains supplementary material available at 10.1186/s12931-022-02166-8.

## Background

The COVID-19 pandemic has affected more than 400 million people to date [1], 20% of whom have been hospitalized [2], underlining the importance of understanding long-term sequelae in survivors. Available data indicate that a third of COVID-19 patients admitted to hospital may progress to acute respiratory distress syndrome (ARDS) [2]. A percentage of ARDS survivors will develop fibrotic pulmonary lesions related to fibroblast accumulation and collagen deposition and other extracellular matrix components [3]. Despite use of protective ventilation protocols, these restrictive changes will reduce quality of life [3, 4] and in some cases result in permanent disabilities. Moreover, a percentage of patients surviving other pneumonias caused by coronavirus such as Severe Acute Respiratory Syndrome (SARS) and Middle East Respiratory Syndrome (MERS), developed long-term functional impairment and fibrotic radiological changes. Older age and male sex were identified as risk factors for unfavorable outcomes and development of pulmonary fibrosis, and correlated with severity and duration of acute illness [5–9].

In the acute phase of COVID-19, mechanical ventilation patient autopsies showed more pronounced hyperplastic and metaplastic changes of pneumocytes and interstitial fibrosis than those receiving conventional oxygen [10]. Early high resolution computed tomography (HRCT) appearance of fibrotic changes have been reported during hospitalization, suggesting that the direct effect of the virus on pulmonary alveolar and endothelial cells combined with an aberrant local immune response could induce pulmonary fibrosis in predisposed patients [11]. Comparing patient HRCT at 60 and 100 days after discharge, there is improvement in the consolidation and extent of ground-glass opacities in images but only a gradual improvement in reticular lesions [12].

Approximately a third of patients with severe pneumonia presented with fibrotic changes, defined as presence of parenchymal bands, traction bronchiectasis and/or honeycombing within 6 months of discharge. In these patients, need for non-IMV was identified as a risk factor [13], and 25% of patients admitted for COVID-19 pneumonia maintained radiological alterations 1 year after discharge, with the most frequent residual alteration (13%) being subpleural reticular/cystic lesions [14].

A recently published study analyzed functional changes over 1 year in severe COVID-19 pneumonia survivors, excluding mechanically ventilated patients. They report pulmonary function improvement at follow-up, but 33% of patients showed diffusing capacity of the lungs for carbon monoxide (DLCO) abnormalities, while fibrotic changes on HRCT were described in less than 5% [15].

We hypothesized that patients with COVID-19 pneumonia may develop pulmonary sequelae regardless of treatment or not with mechanical ventilation. The aim of our study was to investigate persistent fibrotic-like lesions and changes in lung function in a cohort of patients with bilateral COVID-19 pneumonia 1 year after hospital discharge.

## Methods

### Study design and participants

COVID-FIBROTIC (clinicaltrials.gov NCT04409275) is a prospective, multicenter, observational follow-up study of patients admitted for bilateral COVID-19 pneumonia in 12 hospitals in Spain. Diagnosis of severe acute respiratory syndrome coronavirus 2 (SARS-CoV-2) was based on centers of disease control (CDC) criteria, with all patients confirmed by reverse transcription polymerase chain reaction (PCR). Diagnosis of COVID-19 pneumonia was established in accordance with World Health Organization (WHO) interim guidance if patients met any of the following criteria: oxygen saturation (SpO_2_) < 94% in room air at sea level, arterial partial pressure of oxygen to fraction of inspired oxygen (PaO_2_/FiO_2_) ratio < 300 mmHg, respiratory frequency > 30 breaths/min, or lung infiltrates > 50% [16]. The extent of pneumonia at the time of emergency room diagnosis was quantified by adapting the Radiographic Assessment of Lung Edema (RALE) score to COVID-19 (minimum 0–maximum 8) [17]. When there was no prior history of pulmonary diseases (except for asthma or sleep apnea) or uncontrolled cardiac or renal failure, findings were attributed to SARS-CoV-2 infection.

All patients discharged from respiratory services aged over 18 with a life expectancy > 1 year were invited to participate. Patients with unilateral COVID-19 pneumonia, a previous diagnosis of interstitial lung disease (ILD) or chronic obstructive pulmonary disease (COPD) and/or with difficulties in attending the centers for follow-up visits were excluded. Patients who experienced a pulmonary embolism during admission were not excluded if they were properly anticoagulated and had shown embolism resolution in a previous angioCT.

### Pulmonary function testing (PFT) and 6-m walk test (6MWT)

Pulmonary function tests (PFT) were performed in the respiratory functional testing laboratory in all participating centers and included determination of lung volumes [total lung capacity (TLC), residual volume (RV), functional residual capacity (FRC) using plethysmography], and spirometry [forced vital capacity (FVC), forced expiratory volume in 1 s (FEV1) and FEV1/FVC ratio]. DLCO was determined by the single breath technique using an infrared analyzer, correcting for hemoglobin values. All procedures were performed according to American Thoracic Society (ATS) and European Respiratory Society (ERS) guidelines [18–21].

The 6-min walk test (6MWT) measures the distance that a patient can walk quickly on a flat, hard surface in a period of 6 min. It evaluates the global and integrated responses of all systems involved during exercise, including pulmonary and cardiovascular systems, systemic circulation, peripheral circulation, blood, neuromuscular units and muscle metabolism [22, 23].

### Imaging tests

Control chest X-rays (CXRs) were performed in all patients 2 months after discharge using standardized techniques with computed radiography equipment. Pulmonary damage was quantified using the RALE score. HRCT scans (SOMATOM, Siemens, Germany; AQUILION, Toshiba, Japan; OPTIMA, General Electric, USA) were obtained with subjects in the supine position during breath hold at end-inspiration. Axial reconstructions were performed with a slice thickness of 1 mm, with 1 mm increment, 512 mm × 512 mm (resolution 0.625 mm/10). The same protocol was used in each center, adjusted to the different CT machines. HRCT images were evaluated for presence of ground-glass opacities (GGO), consolidations, bronchiectasis, parenchymal bands and reticulations as defined by the Fleischner society glossary of terms [24]. Fibrotic-like changes on HRCT were defined as presence of traction bronchiectasis, parenchymal bands and/or reticular pattern [25, 26]. Experienced chest radiologists in each centers, blinded to all clinical and functional data, evaluated the images. CT scans performed at 1 year were also compared with the 2-month scan.

### Procedures

During the screening visit (30 days after hospital discharge), demographic data (age, sex, ethnicity) and medical history (smoking, hypertension, diabetes, previous respiratory, cardiac or renal history and concomitant medication) were collected. Data associated with the acute episode were also collected (days of symptom onset, intensity of dyspnea, extent of pneumonia on diagnostic X-ray in the emergency room, maximum radiological extent during admission, days of admission, maximum respiratory support and inflammatory laboratory values). All data were retrieved from electronic medical records and de-identified data were entered into an electronic database (Veridata™ EDC). Patients were assessed 2 months after discharge (visit 1, V1) by collecting residual dyspnea using the modified British Medical Research Council (mMRC) [27], CXR and PFT. Patients with impairment in PFT (FVC < 80% without FEV1/FVC < 70 and/or DLCO < 80%) and/or persistent radiological alteration on CXR underwent thoracic HRCT [28]. Functional changes, exercise capacity using the 6MWT and evolution of dyspnea were assessed at 2 (V1), 6 (visit 2, V2) and 12 months (visit 3, V3) after discharge, repeating chest HRCT in patients without complete resolution at 2-month CT scan. No further treatment was indicated.

For further analysis, patients were stratified according to WHO Ordinal Outcomes Scale [29] into three groups, depending on the maximum respiratory support needed:

1. Group 1: hospitalized mild disease (scale 4): hospitalized patients who required supplemental oxygen by mask or nasal prongs.
2. Group 2: hospitalized severe disease (scale 5): hospitalized patients who required non-invasive ventilation (non-IMV) or high flow nasal oxygen cannula (HFNC).
3. Group 3: hospitalized critical  disease (scale 6–7): hospitalized patients who required respiratory support by intubation and invasive mechanical ventilation (IMV).

### Statistical analysis

We followed the Strengthening the Reporting of Observational Studies in Epidemiology (STROBE) [30] guidelines for reporting observational studies. We calculated the required sample size using the ‘nsize’ command in Stata 12.1 based on data published by Hui [31], who describe a proportion of fibrotic radiological abnormalities on chest X-ray on 27.8% patients (SARS survivors in 2003) assuming a maximum error of 5% and 95% confidence.

Qualitative variables were described using frequencies and percentages, and quantitative variables by means and standard deviation. Normality for continuous variables was checked using the Shapiro-Wilks test and if their normal distribution was not confirmed, variables were expressed with median and interquartile range.

Mean comparison was carried out using the Student t-test in the presence of normality, and if otherwise, using the Mann–Whitney U test. For qualitative variables, comparison of percentages between groups was carried out using Fisher's exact test for dichotomous variables or chi-square test for contingency tables with more than two categories. Patient pulmonary function at follow-up was assessed with mixed linear models for quantitative outcomes, with individual identification key as random effect and time and/or severity status and interaction as independent factors. Cochran-Q test was used in the case of dichotomous outcomes. Factors associated with diffusion impairment and fibrotic pattern at 12 months were studied using multivariable logistic regression. The variables for the multivariable analysis were selected using Akaike’s Information Criterion in a backward-forward stepwise procedure.

Effect of time and severity in DLCO, 6MM and FVC was evaluated using linear mixed models with time (V1, V2, V3) and severity (mild, moderate, severe) as fixed effects and individuals as random effect; interaction between severity and time was also included. Compound symmetry was used for correlation between time measures of same individual. Similarly dyspnea was dichotomized into 2 or more versus less than 2, model proposed was a generalized linear mixed model with time, severity and interaction between both fixed effects and individuals as random effect. Post-hoc analysis was carried out in the interaction term with p value adjustment according to Bonferroni method for 36 tests.

## Results

**Fig. 1:**
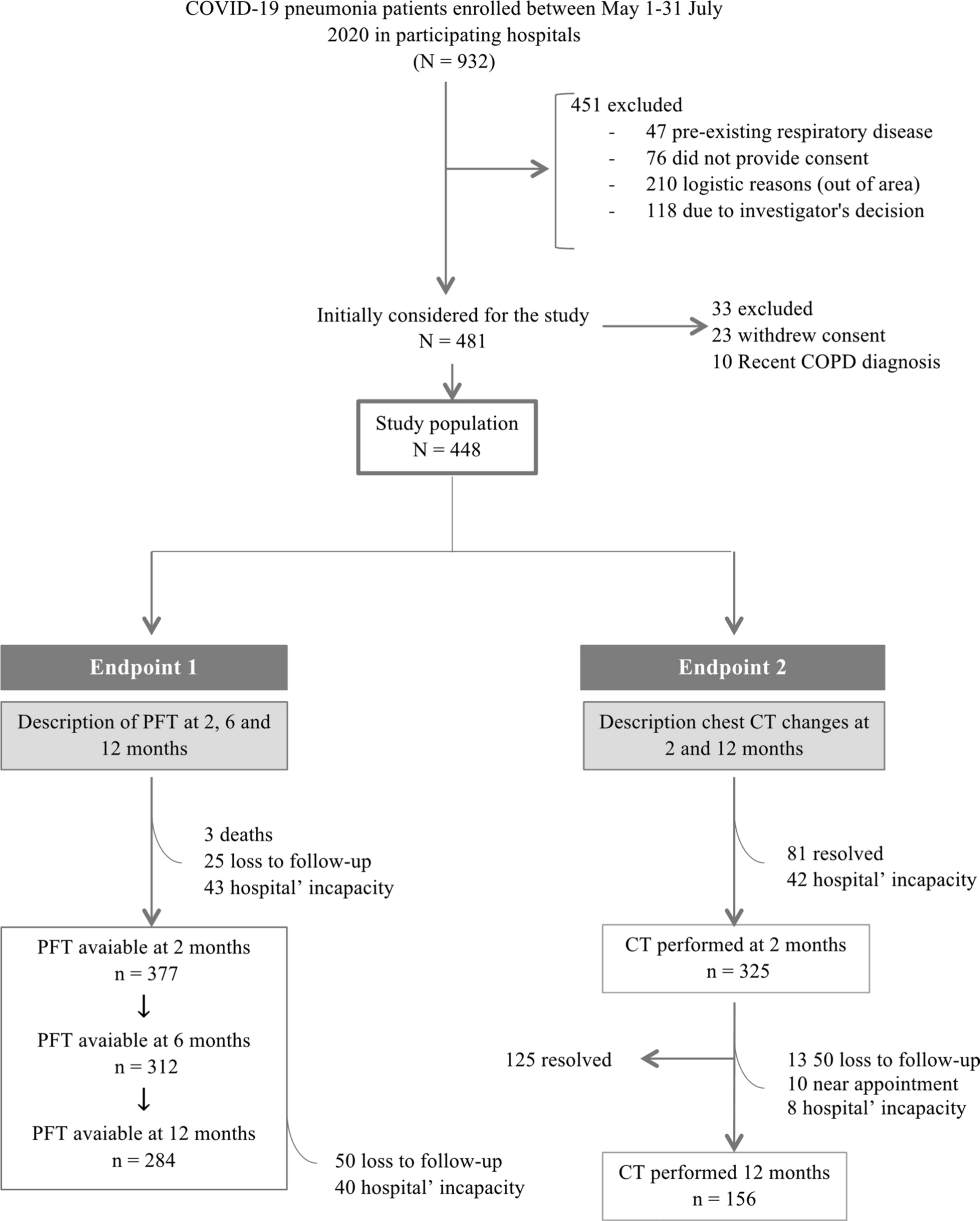
Flow chart of patients discharged from participating hospitals included in the COVID-FIBROTIC cohort. At 2 months a CT scan was ordered when there were alterations at chest radiography and/or pulmonary function test abnormalities. *COPD* chronic obstructive pulmonary disease, *PFT* pulmonary functional test, *CT* computed tomography

Between May 1 and July 31, 2020, 932 patients were attended for COVID-19 pneumonia in participating hospitals, 481 of whom were initially considered for follow-up. At the end of the study, we retrieved full data in 377, 312 and 284 patients at 2, 6 and 12 months, respectively (Fig. 1). Most of the data were lost due to pandemic restrictions.

Analysis of the cohort that completed the 12-month period showed a mean age of 60.5 (11.9) years, and 55.3% (157/284) of them were men, which significantly increased as did severity: 50.2% (105/209) vs. 60.9% (14/23) vs. 73.1% (38/52) [p = 0.010]. We also found between-group differences in length of hospital stay [p < 0.001], RALE scores [p < 0.001] and laboratory parameters (lower lymphocyte count and higher peak levels of lactated dehydrogenase, C-reactive protein, ferritin and D-dimer) [all p < 0.001]. In contrast, there were no significant differences between groups in age, comorbidities, smoking, or body mass index (BMI) (Table 1). The average time to post-hospital discharge appointments was 63 (14) days for V1, 181 (10) for V2 and 365 (17) for V3.

**Table 1:** Characteristics of completed patients

	Severity group 1 n = 209	Severity group 2 n = 23	Severity group 3 n = 52	Total n = 284	*p*-value
Age, years	59.9 (12.5)	61.4 (10.7)	62.7 (9.6)	60.5 (11.9)	0.28
Male sex	105 (50.2%)	14 (60.9%)	38 (73.1%)	157 (55.3%)	0.01
BMI, kg/m^2^	28.0 (4.8)	27.3 (4.1)	28.0 (4.4)	28.0 (4.7)	0.75
Never-smoker	122 (58.4%)	17 (73.9%)	25 (48.1%)	164 (57.7%)	0.14
Comorbidities					
Pulmonary disease^a^	39 (18.7%)	4 (17.4%)	7 (13.5%)	50 (17.6%)	0.69
Hypertension	82 (39.2%)	5 (21.7%)	25 (48.1%)	112 (39.4%)	0.09
Diabetes	30 (14.4%)	1 (4.3%)	10 (19.2%)	41 (14.4%)	0.26
Cardiovascular disease	21 (10.0%)	3 (13%)	4 (7.7%)	28 (9.9%)	0.74
Admission RALE score	3.2 (1.6)	4.1 (1.5)	4.3 (1.7)	3.5 (1.7)	< 0.001
Peak RALE score	3.2 (1.6)	4.2 (1.8)	6.0 (1.6)	6.5 (1.6)	< 0.001
Length of hospital stay, days	9.2 (5.1)	19.9 (6.4)	43.6 (27.5)	16.5 (18.4)	< 0.001
Laboratory findings^b^					
Lymphocytes, × 10^9^/L	1.0 (0.5)	0.7 (0.5)	0.5 (0.3)	0.9 (0.5)	< 0.001
LDH, U/L	514.0 (224.8)	592.5 (221.7)	873.8 (360.1)	590.7 (291.8)	< 0.001
C-reactive protein, mg/L	81.7 (79.9)	222.3 (120.2)	234.5 (145.4)	121.3 (118.4)	< 0.001
Ferritin, ng/mL	838.2 (713.6)	1506.1 (1031.9)	2133.3 (1469.0)	1151.3 (1064.2)	< 0.001
Fibrinogen, g/L	6.4 (1.6)	8.2 (2.2)	21.8 (82.6)	9.5 (36.2)	0.08
D-dimer, ng/mL	1957.6 (4280.8)	3106.3 (4984.6)	10,994.5 (10,535.6)	3631.2 (6796.3)	< 0.001

Data are n (%) or mean (SD). Severity group 1: mild. Severity group 2: moderate. Severity group 3: severe

*BMI* body mass index, *RALE* radiographic assessment of lung edema, *LDH* lactate dehydrogenase

^a^Pulmonary disease: asthma, obstructive sleep apnea

^b^All laboratory findings are peak values except for lymphocytes which is the lowest value

### Pulmonary function tests

**Fig. 2:**
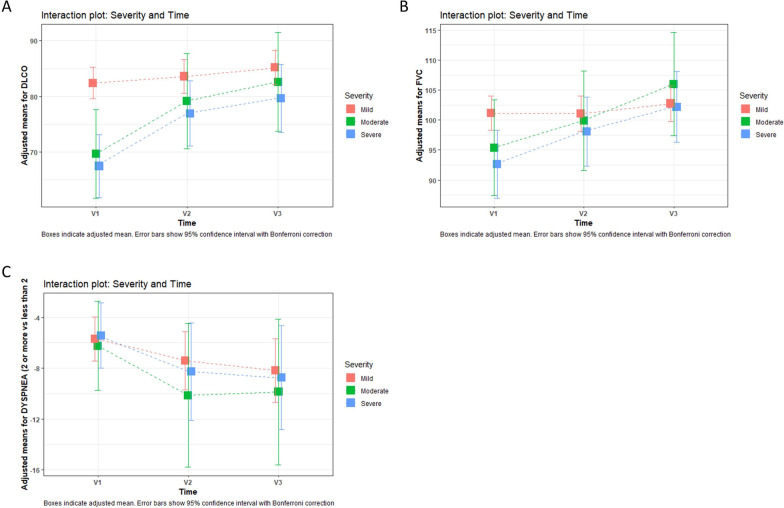
Interaction plot of severity and time based on linear mixed model post-hoc analysis. Boxes indicate adjusted mean. Error bars show 95% confidence interval with Bonferroni correction. V1 (2 months), V2 (6 months) and V3 (12 months). Group 1: mild; group 2: moderate; group 3: severe. **A** Interaction plot: changes over time and severity in % of predicted D_LCO_. We found differences between mind and moderate [p = 0.001] or severe [p < 0.001] patients only at 2 months (V1). **B** Interaction plot: changes over time and severity in FVC% of predicted. No between-group differences were found at any time. **C** Interaction plot: changes over time and severity in dyspnea. No between-group differences were found at any time. *FVC* forced vital capacity, *D*_*LCO*_ diffusing capacity of lungs for carbon monoxide

Analyzing pulmonary function tests from the whole cohort, 53.8% (203/377) of patients had diffusion impairment (< 80% of predicted DLCO) at 60 days, with an improving trend at 180 and 365 days (46.8% [146/312] and 39.8% [113/284], respectively) [p < 0.001]. The mean (% of predicted) DLCO in V1, V2 and V3 was 78.5 (19.1), 81.6 (16.4) and 84 (16.4), respectively [p < 0.001] (Table 2). However, turning to changes in diffusion (% of predicted DLCO) as a function of severity (groups 1, 2 and 3) and dynamics over time, we only found significant differences between mild and moderate [p = 0.001] or severe [p < 0.001] patients at 2 months (V1) (Fig. 2A).

**Table 2:** Pulmonary function test of patients at follow-up

	First follow-up (2 months) n = 377	Second follow-up (6 months) n = 312	Third follow-up (12 months) n = 284	*p*-value
FVC, L	3.50 (1.08)	3.55 (1.02)	3.66 (1.05)	< 0.001
FVC, % pred	98.99 (17.86)	100.76 (16.49)	104.16 (16.10)	< 0.001
FVC < 80%, pred	54 (14.32%)	29 (9.29%)	19 (6.69%)	< 0.001
FEV1, L	2.79 (0.86)	2.81 (0.82)	2.89 (0.84)	< 0.001
FEV1, % pred	98.58 (16.93)	100.12 (16.07)	103.53 (15.63)	< 0.001
FEV1/FVC	79.58 (6.87)	79.03 (6.30)	78.75 (5.92)	0.004
D_LCO_, % pred	78.47 (19.14)	81.61 (16.37)	84.03 (16.37)	< 0.001
D_LCO_ < 80%, pred	203 (53.80%)	146 (46.79%)	113 (39.78%)	< 0.001
D_LCO_/V_A_, % pred	92.55 (17.37)	95.20 (17.25)	95.66 (17.16)	0.001
D_LCO_/V_A_ < 80%, pred	83 (22.02%)	57 (18.26%)	48 (16.90%)	0.02
TLC, % pred	95.61 (14.90)	96.47 (14.33)	96.88 (14.51)	0.08
RV, % pred	96.90 (23.65)	96.75 (23.18)	95.02 (23.80)	0.72
6MWT distance, m	523.63 (94.30)	520.57 (104.53)	518.82 (101.66)	0.004
mMRC^a^				
0–1	216 (78.54%)	244 (88.72%)	248 (90.18%)	< 0.001
≥ 2	59 (21.45%)	31 (11.27%)	27 (9.82%)	

Data are n (%) or mean (SD)

*FVC* forced vital capacity, *FEV1* forced expiratory volume in one second, *D*_*LCO*_ diffusing capacity for carbon monoxide, *V*_*A*_ alveolar volume, *TLC* total lung capacity, *RV* residual volume, *6MWT* 6 min walk test (m), *mMRC* modified British Medical Research Council (dyspnea)

^a^Dyspnea was registered on 275 patients

Restrictive abnormality (FVC < 80% of predicted) across the whole cohort was present in 14.3% [54/377], 9.3% [29/312] and 6.7% [19/284] of patients at 2, 6 and 12 months, respectively [p = 0.001] and mean FVC (% of predicted) was 99 (17.9), 100.8 (16.5) and 104.2 [16], at 2, 6 and 12 months [p < 0.001] (Table 2). Nevertheless, we did not found significant differences when we analyzed FVC (% of predicted) as a function of severity and time (Fig. 2B).

We next analyzed dyspnea across the entire cohort. Dyspnea ≥ 2 at mMRC scale was 21.5% [59/275], 11.3% [31/275] and 9.8% [27/275] at 2, 6 and 12 months (Table 2) and association between dyspnea and follow-ups was found to be significant [p < 0.001]. However, when evaluating the possible changes between severity groups during follow-up, no significant differences were found (Fig. 2C).

We did not found differences between-groups on static volumes and, although we found differences at 6MWT, a decrease in the distance walked was obtained (mean 523.6 vs. 520.6 vs. 518.8; [p = 0.004]) (Table 2). However, severity might be a confounding factor because no significant trend was found when adjusting by it (Additional file 1: Fig. S1).

Associated factors with altered DLCO < 80% at 12 months according to multivariable regression were age (OR 1.04, 95% CI 1.00–1.07, p = 0.013), female sex (OR: 6.22, 95% CI 2.77–15.04, p = 0.001), BMI (OR: 0.91, 95% CI 0.84–0.99, p = 0.001) and ferritin levels (OR: 1.00, 95% CI 1.00–1.01, p = 0.008) (Table 3).

**Table 3:** Multivariate logistic regression to predict the diffusion impairment at 12 months

	Total N	Missing	Univariable analysis		Multivariable analysis n = 144	
			OR (95% CI)	*p*-value	OR (95% CI)	*p*-value
Age, years	283	1	1.02 (1.00–1.04)	0.01	1.04 (1.00–1.07)	0.013
Female sex	284	0	2.53 (1.56–4.14)	0.001	6.22 (2.77–15.04)	0.001
BMI, kg/m^2^	278	6	0.98 (0.93–1.03)	0.48	0.91 (0.84–0.99)	0.036
Smoker	283	1	1.48 (0.91–2.43)	0.11	N/A	N/A
Pulmonary disease	284	0	0.66 (0.34–1.25)	0.21	N/A	N/A
Hypertension	284	0	1.02 (0.63–1.66)	0.91	N/A	N/A
Diabetes	284	0	1.08 (0.54–2.11)	0.81	N/A	N/A
Cardiovascular disease	284	0	2.18 (0.99–4.91)	0.05	NA	NA
Admission RALE score	278	6	1.08 (0.94–1.25)	0.24	N/A	N/A
Peak RALE score	278	6	1.07 (0.94–1.21)	0.25	N/A	N/A
Length of hospital stay, days	280	4	1.01 (1.00–1.03)	0.01	NA	NA
Lymphocytes^a^, × 10^9^/L	240	44	0.84 (0.49–1.40)	0.51	N/A	N/A
LDH, U/L	241	43	1.00 (1.00–1.01)	0.02	NA	NA
C-reactive protein, mg/L	249	35	1.00 (0.99–1.00)	0.14	N/A	N/A
Ferritin, ng/mL	204	80	1.00 (0.99–1.00)	0.15	1.00 (1.00–1.01)	0.008
Fibrinogen, g/L	178	106	1.07 (1.00–1.27)	0.39	1.02 (0.99–1.26)	0.79
D-dimer, ng/mL	252	32	1.00 (1.00–1.01)	0.01	1.00 (1.00–1.01)	0.07
Severity group 3	284	0	2.25 (1.22–4.20)	0.009	1.05 (0.33–3.33)	NA

*OR* odds ratio, *CI* confidence interval, *BMI* body mass index, *RALE* radiographic assessment of lung edema, *LDH* lactated dehydrogenase

^a^All laboratory findings are peak values except for lymphocytes which is the lowest value

Variables selected according to backward-forward stepwise selection AIC

### Radiological findings

According to the study protocol, chest CT was indicated 2 months after discharge in patients with persistent dyspnea, pulmonary function test alteration or abnormal chest radiography. At V1, we performed HRCT on 325 patients, 38.4% of whom showed complete resolution (125/325). In the remaining 200 patients, the most frequent radiological pattern was GGO, reported in 73.5% [147/200] (32% when we consider the study cohort [147/448]), with between-group differences (Additional file 2: Table S1). According to protocol, patients underwent a new chest CT 1 year after discharge if abnormal changes had been present in the previous one. Finally, 156 patients out of 200 (78%) had CT at 12 months (Fig. 1) and any radiological alteration persisted in 78.8% of patients with a second CT [123/156], 27.4% when considering our study cohort [123/448]. Regarding these CT abnormalities, GGO was found in 45.5% of the performed CT [71/156] (or 15.8% on the study cohort [71/448]), reticular pattern in 34% [53/156] (11.8% [53/448]); traction bronchiectasis in 30.8% [48/156] (10.7% [48/448]) and parenchymal bands in 33.4% [52/156] (11.6% [52/448]). In total, fibrotic-like sequelae were found at 12 months in 65.4% of the performed CT [102/156], 22.7% when considering our study cohort [102/448] (Table 4). In addition, these changes were more frequent among more severe patients, with significant between-group differences [p = 0.001].

**Table 4:** Chest CT at 12-month follow-up according to severity groups

	Severity group 1 n = 81	Severity group 2 n = 18	Severity group 3 n = 57	Total n = 156	*p*-value
Normal CT pattern	28 (34.6%)	2 (11.1%)	3 (5.3%)	33 (21.2%)	0.001
Consolidation	14 (17.3%)	0 (0%)	11 (19.3%)	25 (16%)	0.12
GGO	29 (35.8%)	11 (61.1%)	31 (54.4%)	71 (45.5%)	0.02
Reticular pattern	26 (32.1%)	4 (22.2%)	23 (40.4%)	53 (33.9%)	0.36
Traction bronchiectasis	14 (17.3%)	6 (33.3%)	28 (49.1%)	48 (30.8%)	0.001
Parenchymal bands	14 (17.3%)	6 (33.3%)	32 (56.1%)	52 (33.4%)	0.001
Fibrosis-like changes^a^	39 (48.1%)	15 (83.4%)	48 (84.2%)	102 (65.4%)	0.001

Data are n (%). Severity group 1: mild. Severity group 2: moderate. Severity group 3: severe

*CT* computed tomography, *GGO* ground glass opacity

^a^Fibrotic-like changes: defined as the presence of traction bronchiectasis, reticular pattern and/or parenchymal bands

The only factors associated with fibrotic pattern at 1 year according to multivariable regression were radiological involvement at admission (OR: 1.55, 95% CI 1.06–2.38; p = 0.02) and LDH (OR: 0.99, 95% CI 0.98–0.99; p = 0.046) (Table 5).

**Table 5:** Multivariable logistic regression to predict the fibrotic-like impairment at 12 months

	Total N	Missing	Univariable analysis		Multivariable analysis n = 80	
			OR (95% CI)	*p*-value	OR (95% CI)	*p*-value
Age, years	156	0	1.01 (0.98–1.05)	0.37	0.99 (0.93–1.05)	0.77
Female sex	156	0	0.50 (0.25–1.00)	0.05	N/A	N/A
BMI, kg/m^2^	144	12	0.93 (0.85–1.00)	0.08	N/A	N/A
Smoker	155	1	1.32 (0.68–2.61)	0.40	N/A	N/A
Pulmonary disease	156	0	0.93 (0.38–2.35)	0.87	2.08 (0.49–9.68)	0.32
Hypertension	156	0	0.94 (0.48–1.85)	0.87	1.13 (0.34–3.69)	0.83
Diabetes	156	0	0.95 (0.42–2.23)	0.90	N/A	N/A
Cardiovascular disease	156	0	2.18 (0.99–4.91)	0.05	NA	NA
Admission RALE score	154	2	1.21 (0.99–1.49)	0.05	1.55 (1.06–2.38)	0.02
Peak RALE score	154	2	1.26 (1.05–1.53)	0.01	0.67 (0.41–1.04)	0.08
Length of hospital stay, days	153	3	1.04 (1.01–1.06)	0.001	NA	NA
Lymphocytes^a^, × 10^9^/L	140	16	0.41 (0.19–0.77)	0.01	0.28 (0.07–0.91)	0.053
LDH, U/L	132	24	1.00 (0.99–1.00)	0.66	0.99 (0.98–0.99)	0.046
C-reactive protein, mg/L	139	17	1.00 (1.00–1.01)	0.001	1.00 (0.99–1.00)	0.18
Ferritin, ng/mL	114	42	1.00 (1.00–1.01)	0.03	NA	NA
Fibrinogen, g/L	104	52	0.99 (0.99–1.00)	0.47	0.99 (0.99–1.00)	0.18
D-dimer, ng/mL	138	18	1.00 (1.00–1.01)	0.005	1.00 (1.00–1.01)	0.09
Severity group 3	156	0	5.74 (2.58–13.19)	0.001	3.38 (0.66–19.81)	0.15

Variables selected according to backward-forward stepwise selection AIC

*OR* odds ratio, *CI* confidence interval, *BMI* body mass index, *RALE* radiographic assessment of lung edema, *LDH* lactated dehydrogenase

^a^All laboratory findings are peak values except for lymphocytes which is the lowest value

## Discussion

In this multicenter prospective study of hospitalized patients with bilateral COVID-19 pneumonia of variable severity, we analyzed functional and radiological sequelae 12 months after hospital discharge, and we have found that reduced lung diffusion persisted in almost 40% of patients (DLCO < 80%) 1 year after acute COVID 19 infection. Furthermore, we identified radiological fibrotic-like changes on chest CT in almost 23% of our study cohort [n = 448]. However, these sequelae were not associated with other markers of severity previously described in ARDS cases [32], such as need for mechanical ventilation.

Along with an increasing number of people affected over time, survival has improved since the start of the pandemic [33], but with the consequence that millions of survivors could be affected by pulmonary sequelae of COVID-19, which could lead to a clear deterioration in quality of life.

Although functional recovery from severe COVID-19 pneumonia occurs within 1 year after discharge, approximately one third of patients (39.8%) still have decreased DLCO. These findings are in line with the results from a Chinese cohort [15], albeit excluding patients requiring invasive mechanical ventilation, and are consistent with a recently published meta-analysis [34]. Among our key findings is that although more severe patients showed higher diffusion impairment 2 months after discharge, this difference lost significance at 6 and 12 months. Furthermore, analyzing factors related to the persistence of DLCO deterioration, 1 year after discharge, we found association with age, female sex, body mass index and ferritin, but need of invasive mechanical ventilation (IMV) (most severe disease) was surprisingly not. The association between female sex and DLCO disturbances is consistent with previously published results at 6 [35, 36] and 12 months in COVID-19 pneumonia survivors [15], with no clear explanation forthcoming.

A recent systematic review showed that histopathological findings of diffuse alveolar damage caused by COVID-19 are indistinguishable from those provoked by other causes. At final, chronic/fibrotic phase was identified, showing a honeycomb lung with collagen fibrosis of the alveolar spaces and an interstitium with thickening of the alveolar wall together with squamous metaplasia of the alveoli [37]. These changes have been reported in 43% of 30 COVID-19 autopsies, and were associated with longer duration of illness and hospitalization and need for mechanical ventilation [38].

The ARDS repair process involves rapid fibroblast proliferation and this leads to extracellular matrix deposition [3], which in a number of patients will remain, resulting in residual fibrosis. However, our group found an increase in serum biomarkers of pulmonary fibrosis (MMP7, MMP1, and periostin) in patients with early fibrotic changes in chest CT at 2 months after hospital discharge [36], not only in IMV patients but also in those treated with conventional or high flow nasal cannula oxygen. A number of environmental and patient-specific factors may also contribute to this fibroproliferation.

In our study cohort, any radiological abnormality at 12 months persisted in 27% [123/448] of patients. This percentage is similar to that obtained by Pan et al. (53/209) [14] and somewhat higher than that recently published by Wu et al. (24%), although patients with invasive mechanical ventilation were excluded in their cohort [15].

We define fibrotic-like changes as the presence of traction bronchiectasis, parenchymal bands and/or reticular pattern [25, 26, 39]. The analysis of fibrotic-like changes in our study population yielded results (23% [102/448]) similar to those found by a recent systematic review reporting radiological fibrotic-like sequelae in 21% of patients 1 year after discharge [40], although other meta-analyses have detected a higher percentage (29%) [34]. In our study, fibrotic-like changes were only associated with radiological involvement at admission and peak LDH levels. Although more severe patients (requiring IMV) had a significantly higher percentage of fibrotic sequelae than the mild group (84.2% [48/57] vs. 48.1% [39/81]), no significance was found between severity and fibrotic-like changes in the multivariable model [p = 0.15].

Mechanical ventilation is a recognized factor in fibrosis development [32], caused by mechanical stress and an induced “biotraumatic” inflammatory response involving cytokine, chemokine and growth factor release. This also supports our finding that radiological abnormalities were most common in the severe group. However, assessment of fibrotic sequelae after COVID-19 infections without taking mechanical ventilation into account could reveal changes directly induced by the effect of the virus. A recent study showed that 4.8% of mild patients had inflammatory interstitial lung disease at 3 months [41], and at 6 months Han et al. detected radiological alterations after discharge in 114 patients, of which only four had required invasive mechanical ventilation [13]. Biomedical research proposes models in which the injury underlying viral infection in the lung induces fibrosis by various mechanisms. As mentioned, the elevated fibrogenesis-related biomarkers seen in these patients indicate that bilateral COVID-19 pneumonia may trigger certain biological pathways [36]. The SARS-CoV-2 infection damages the alveolar epithelium and induces cytokine production; this attracts macrophages that contribute to basement membrane damage and fibroblast activation. Furthermore, hemorrhage due to endothelial injury activates a coagulation cascade that culminates in fibrin deposition. All this contributes to fibrosis of the alveolar space [42].

Whether these changes involve scarring from the acute process or whether they might progress over time is still unclear [43]. Although the virus is eradicated in COVID-19 recovered patients, removing the cause of lung damage does not in itself preclude development of irreversible progressive and fibrotic interstitial lung disease [44].

This study has several limitations. Firstly, data on functional tests or CT scans prior to admission were not available for assessment of longitudinal changes. Secondly, following the study protocol, chest CT was performed initially only in patients with radiological or functional changes at 2 months, and at 12 months when initial CT was altered. This approach aimed to minimize radiological exposure and overload in radiology services during the pandemic, and follows guidelines recommended by several societies [28, 45]. In a small percentage of patients without previous resolution, chest CT at 1 year was not available, so the percentage of fibrotic sequelae could potentially be underestimated. Nonetheless, excluding patients with previous pulmonary interstitial disease or chronic obstructive pulmonary disease, and the fact that these are asymptomatic patients with no functional or chest X-ray alterations after discharge, seem unlikely to result in significant radiological alterations. Likewise, altered DLCO has been described in 98% of patients with fibrosis at the time of initial evaluation, so the percentage of losses in cases of normal DLCO would potentially be low [46]. Another important limitation has been the loss of patients due to pandemic-related restrictions and security measures in pulmonary function laboratories. However, no differences were found between patients who underwent all tests compared to those who did not (Additional file 2: Table S2).

## Conclusions

Approximately one third of patients who survived a severe COVID-19 pneumonia had impaired lung function and dyspnea 12 months after hospital discharge, and 23% developed fibrotic-like sequelae, in both severe and mild patients. These findings confirm the need for follow-up of patients with severe SARS-CoV-2-induced pneumonia to clarify whether fibrotic changes may progress over time.

## Supplementary Information

**Additional file 1: Figure S1.** Interaction plot severity and time in 6MWT based on linear mixed model post-hoc analysis. V1 (2 months), V2 (6 months) and V3 (12 months). Group 1: mild; group 2: moderate; group 3: severe. No between-group differences were found at any time. 6MWT = 6 min walk test.

**Additional file 2: Table S1.** Chest CT at 2-month follow-up according to severity group. **Table S2.** Table for reviewer. Characteristics of enrolled patients vs. lost patients.

## Data Availability

The datasets used and analyzed during the current study are available from the corresponding author on reasonable request.

## Abbreviations

COVID-19: Coronavirus disease 2019
ARDS: Acute respiratory distress síndrome
SARS: Severe acute respiratory syndrome
MERS: Middle east respiratory síndrome
HRCT: High resolution computed tomography
non-IMV: Non invasive mechanical ventilation
DLCO: Diffusing capacity for carbon monoxide
SARS-CoV-2: Severe acute respiratory syndrome coronavirus 2
PCR: Polymerase chain reaction
CDC: Centers of disease control
WHO: World Health Organization
SpO: Oxygen saturation
PaO_2_/FiO_2_: Rterial partial pressure of oxygen to fraction of inspired oxygen
ILD: Interstitial lung disease
COPD: Chronic obstructive pulmonary disease
angioCT: Vascular computed tomography
PFT: Pulmonary function test
6-MWT: 6 min-walk test
TLC: Total lung capacity
RV: Residual volumen
FRC: Functional residual capacity
FVC: Forced vital capacity
FEV1: Forced expiratory volume in 1 s
ATS: American Thoracic Society
ERS: European Respiratory Society
CXRs: Chest X-rays
RALE: Radiographic assessment of lung edema
CT: Computed tomography
GGO: Ground-glass opacity
mMRC: Modified British Medical Research Council
V1: Visit 1
V2: Visit 2
V3: Visit 3
HFNC: High flow nasal cannula oxygen
IMV: Invasive mechanical ventilation
STROBE: Strengthening the Reporting of Observational Studies in Epidemiology
BMI: Body mass index
LDH: Lactated dehydrogenase
MMP: Matrix metalloproteinases
