# Supplementary figures and images for: Lung function and radiological findings 1 year after COVID-19: a prospective follow-up
Source: Respir Res. 2022 Sep 12;23:242. doi: 10.1186/s12931-022-02166-8 (PMC9466319; doi:10.1186/s12931-022-02166-8)

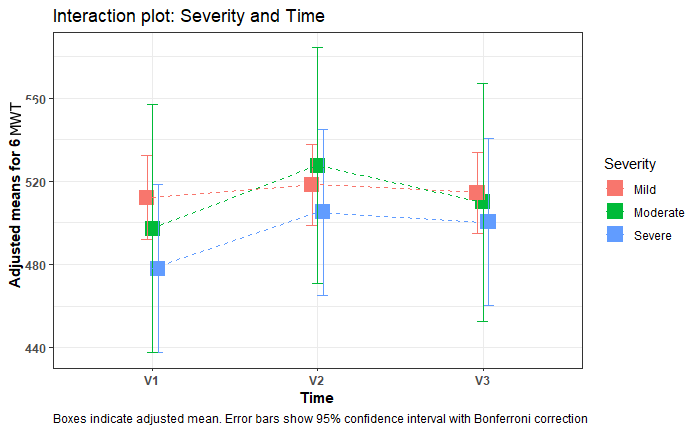

Supplement: Supplementary file 1 — Additional file 1: Figure S1. Interaction plot severity and time in 6MWT based on linear mixed model post-hoc analysis. V1 (2 months), V2 (6 months) and V3 (12 months). Group 1: mild; group 2: moderate; group 3: severe. No between-group differences were found at any time. 6MWT = 6 min walk test. [file 12931_2022_2166_MOESM1_ESM.png]
