# Supplementary material for: Lung function and radiological findings 1 year after COVID-19: a prospective follow-up
Source: Respir Res. 2022 Sep 12;23:242. doi: 10.1186/s12931-022-02166-8 (PMC9466319; doi:10.1186/s12931-022-02166-8)
Supplement: Supplementary file 2 — Additional file 2: Table S1. Chest CT at 2-month follow-up according to severity group. Table S2. Table for reviewer. Characteristics of enrolled patients vs. lost patients. [file 12931_2022_2166_MOESM2_ESM.docx]

ADDITIONAL TABLES

Table S1: Chest CT at 2-month follow-up according to severity group

|  | **Severity Group 1**  **n = 209** | **Severity Group 2**  **n = 32** | **Severity Group 3**  **n = 84** | **Total**  **n = 325** | ***p*-value** |
| --- | --- | --- | --- | --- | --- |
| Normal CT pattern | 102 (48.8%) | 7 (21.9%) | 16 (19%) | 125 (38.4%) | 0.001 |
| Consolidation | 17 (8.1%) | 2 (6.2%) | 14 (16.7%) | 33 (10.2%) | 0.085 |
| GGO | 77 (36.8%) | 23 (71.9%) | 47 (56.0%) | 147 (45.2%) | 0.001 |
| Reticular pattern | 24 (11.5%) | 5 (15.6%) | 28 (33.3%) | 57 (17.5%) | 0.001 |
| Traction Bronchiectasis | 17 (8.1%) | 9 (28.1%) | 29 (34.5%) | 55 (16.9%) | 0.001 |
| Parenchymal bands | 24 (11.5%) | 7 (21.9%) | 27 (32.1%) | 58 (17.8%) | 0.001 |

Data are n (%). Severity Group 1: mild. Severity Group 2: moderate. Severity Group 3: severe.

CT= computed tomography. GGO= ground glass opacity.

Table S2: Table for reviewer. Characteristics of enrolled patients vs. lost patients

|  | **Enrolled patients**  **n = 284** | **Lost patients**  **n = 164** | ***p* Value** |
| --- | --- | --- | --- |
| Age, years | 60.5 (11.9) | 60.8 (14.4) | 0.80 |
| Male sex | 157 (55.3%) | 102 (62.2%) | 0.18 |
| BMI, kg/m^2^ | 28.0 (4.7) | 29.3 (4.9) | 0.006 |
| Never-smoker | 164 (57.7%) | 89 (54.3%) | 0.76 |
| Comorbidities |  |  |  |
| Pulmonary disease* | 50 (17.6%) | 27 (16.5%) | 0.88 |
| Hypertension | 112 (39.4%) | 73 (44.5%) | 0.31 |
| Diabetes | 41 (14.4%) | 39 (23.8%) | 0.017 |
| Cardiovascular disease | 28 (9.9%) | 20 (12.2%) | 0.52 |
| Admission RALE score | 3.5 (1.7) | 3.8 (1.9) | 0.084 |
| Peak RALE score | 4.8 (2.0) | 5.2 (2.0) | 0.062 |
| Length of hospital stay, days | 16.5 (18.4) | 19.7 (16.2) | 0.065 |
| Severity |  |  |  |
| Group 1 (mild) | 209 (73.6%) | 105 (64%) | 0.10 |
| Group 2 (moderate) | 23 (8.1%) | 19 (11.6%) |  |
| Group 1 (severe) | 52 (18.3%) | 40 (24.4%) |  |

Data are n (%) or mean (SD). BMI= body mass index. RALE= radiographic assessment of lung edema. *Pulmonary disease: asthma, obstructive sleep apnea, other.
